# Effect of Cnidii Rhizoma on nitric oxide production and invasion of human colorectal adenocarcinoma HT-29 cells

**DOI:** 10.3892/ol.2014.2660

**Published:** 2014-11-03

**Authors:** KYUNG-SOO NAM, BYUNG GEUN HA, YUN-HEE SHON

**Affiliations:** 1 Department of Pharmacology, College of Medicine, Dongguk University, Gyeongju, Gyeongsangbuk-do 780-714, Republic of Korea; 2 Bio-Medical Research Institute, Kyungpook National University Hospital, Daegu 700-721, Republic of Korea

**Keywords:** colorectal adenocarcinoma HT-29 cells, invasion, Cnidii Rhizoma, nitric oxide, matrix metalloproteinase-2, pro-inflammatory cytokines

## Abstract

Colorectal adenocarcinoma is the most common type of gastrointestinal cancer. Colon adenocarcinoma is a major health problem worldwide due to the high prevalence and mortality rates associated with the disease. The majority of colorectal carcinomas are adenocarcinomas, which originate from the epithelial cells of the colorectal mucosa. HT-29 cells, which originate from human colon adenocarcinoma, are used as an *in vitro* model to investigate the effect of malignant transformation on the expression of cellular constituents and functions of the intestinal epithelium. Nitric oxide (NO) is a signaling molecule, which is involved in inflammation and carcinogenesis. It has been reported that enhanced inducible NO synthase (iNOS) activity and the resulting NO concentrations in human colon carcinoma contribute to tumor progression and vascular invasion. The present study investigates the effect of pro-inflammatory cytokine-induced nitric oxide (NO) production and iNOS expression on the invasion of human colorectal adenocarcinoma HT-29 cells, and the effect of extract from Cnidii Rhizoma on NO production and the invasiveness of HT-29 cells. Treatment of HT-29 cells with cytokines, 100 U/ml interferon γ, 10 ng/ml interleukin-1 α and 25 ng/ml tumor necrosis factor α was found to increase NO production. Pretreatment of the cells with Cnidii Rhizoma (0.1–5 mg/ml) resulted in an inhibition of cytokine-induced NO production and iNOS expression. The invasiveness of HT-29 cells through Matrigel was significantly increased by treatment with cytokines. Cnidii Rhizoma inhibited the invasiveness of cytokine-treated HT-29 cells through the Matrigel-coated membrane in a concentration-dependent manner. Matrix metalloproteinase (MMP) activity in HT-29 cells increased following the treatment with cytokines, and pretreatment of the cells with Cnidii Rhizoma inhibited cytokine-induced MMP-2 activity. These results provide sufficient information for the further development of Cnidii Rhizoma as an antitumor metastatic agent for the treatment of colon cancer.

## Introduction

Nitric oxide (NO) is an important bioactive signaling molecule that is significant in numerous physiological processes in the cardiovascular, neurological and immune systems. However, increased NO production may also contribute to the pathogenesis of a variety of disorders, including various cancers, such as breast, cervical, gastric, colorectal and head and neck cancers (1). The formation of NO from arginine is catalyzed by three types of NO synthase (NOS): Endothelial NOS (eNOS), neuronal NOS (nNOS) and the inducible isoenzyme (iNOS) (2). iNOS expression is generally induced by inflammatory stimuli and is responsible for the production of large quantities of NO. It has been reported that the synthesis of NO is induced by cytokines in certain human carcinoma cell lines (3). A previous study has suggested that a high expression of iNOS is associated with the aggressive behavior of colorectal adenocarcinomas (4), however, the biological significance of NO in malignant tumors remains unclear.

Cancer cell invasion and metastasis are complex multi-step processes that involve cell adhesion, degradation of the extracellular matrix by proteolytic enzymes and motility factors that influence cell migration (5). Matrix metalloproteinases (MMPs) are significant in the degradation of the extracellular matrix and the MMP family consists of >20 proteolytic enzymes (6). MMP production appears to be a marker for cancer cells with elevated metastatic potential (7) and the activation of MMP activity has been detected in colon carcinoma (8).

Cnidii Rhizoma is the dried root of *Cnidium officinale* Makino and has been reported to exhibit antitumor activity in ddY mice (9), inhibit liver and lung metastasis of tumor cells *in vivo* (10) and exhibit anti-angiogenic activity in renal glomerular capillary endothelial cells, chick embryo chorioallantoic membrane and rat cornea (11).

N-(3-(aminomethyl)benzyl)acetamidine (1400W), a nontoxic novel NOS inhibitor, is the most selective inhibitor of iNOS (12). 1400W has been reported to be effictive in the treatment of colonic injury in an experimental model of colitis in rats (13). Recently, the potency and selectivity of 1400W, as an inhibitor of iNOS and cytokine release modifier, have indicated a potential use for 1400W in cancer therapy (14).

Colorectal cancer is the second most common cause of cancer in women (9.2% of diagnoses) and the third most common in men (10.0%) worldwide (15). It is a multifactorial disease etiology, which includes genetic factors, environmental exposures, such as diet, and inflammatory conditions of the digestive tract. In Western Europe and the USA the most common type of colon cancer is adenocarcinoma, which accounts for 98% of all cases. Lymphoma and squamous cell carcinoma occur less frequently (16). Adenocarcinoma is a malignant epithelial tumor, originating from the superficial glandular epithelial cells lining the colon and rectum. Conventional adenocarcinoma is characterized by glandular formation, which is the basis for histological tumor grading (17).

The present study investigates the ability of pro-inflammatory cytokine-induced NO to modulate the invasiveness of human colorectal adenocarcinoma HT-29 cells, which is a cell line mainly used as an *in vitro* colon epithelial cell model to investigate absorption, transport and secretion by intestinal cells, and the effect of the extract from Cnidii Rhizoma on NO production and invasiveness of HT-29 cells.

## Materials and methods

### Preparation of Cnidii Rhizoma extract

*Cnidium officinale* Makino root was collected in Jeong-seon, Republic of Korea. Specimens (no. 00C-37) were preserved by air-drying the roots and were deposited in the herbarium of the Intractable Disease Research Center (Dongguk University, Gyeongju, Republic of Korea). Cnidii Rhizoma (60 g) was extracted using 400 ml distilled water for 3 h. The extract was filtered and the 200 ml filtrate was concentrated *in vacuo,* lyophilized using a Freezezone Console Freeze Dry System (7755040; Labconco, Kansas City, MO, USA) and stored at −20°C prior to use. The mean yield of extract was 6.9% of the dried ingredient weight.

### Cell culture

The HT-29 human colon adenocarcinoma cell line (American Type Culture Collection, Manassas, VA, USA) was cultured at 37°C in a humidified atmosphere of 5% CO_2_ in RPMI-1640 medium (Gibco-BRL, Carlsbad, CA, USA), supplemented with 10% (v/v) fetal bovine serum (Gibco-BRL).

### iNOS induction

To induce iNOS expression, subconfluent monolayers were cultured in serum-free medium for 24 h. Growth-arrested cultures were treated with pro-inflammatory cytokines, 100 U/ml interferon γ (IFN-γ) (Sigma-Aldrich, St. Louis, MO, USA), 10 ng/ml interleukin-1 α (IL-1α) (PeproTech, Inc., Rocky Hill, NJ, USA) and 25 ng/ml tumor necrosis factor-α (TNF-α) (R&D Systems, Minneapolis, MN, USA), pro-inflammatory cytokines and 0.1–5 mg/ml water extract of Cnidii Rhizoma or 0.5 mM 1400W (Sigma-Aldrich) in fresh medium without fetal bovine serum. After 48 h, the supernatants were collected and the cells were harvested and lysed as previously described (18).

### Nitrite assay

Nitrite, a stable-end product of NO production in HT-29 cells, was measured as previously described (19) in the supernatants obtained from the cell culture. The protein concentration of the supernatant was determined using a bicinchoninic acid protein assay kit (Sigma-Aldrich) with bovine serum albumin as the standard.

### Western blot analysis

Using a 7% SDS-polyacrylamide gel, electrophoresis was performed to analyze the protein from cell lysates and subsequently electrophoretically transferred to a polyvinylidene difluoride membrane. The membrane was treated with 5% non-fat milk for 1 h to block non-specific binding and probed with a rabbit anti-human polyclonal iNOS antibody (sc-651; Santa Cruz Biotechnology, Santa Cruz, CA, USA) at a final dilution of 1:1,000. The primary antibodies were detected using biotin-rabbit anti-mouse immunoglobulins G, A and M (heavy and light chains; Zymed, San Francisco, CA, USA) and alkaline phosphate-conjugated streptavidin, and were visualized using 4-nitro blue tetrazolium chloride or 5-bromo-4-chloro-3-indolyl-phosphate substrate (Promega, Madison, WI, USA).

### Invasion assay

Cell migration through Matrigel-coated filters was measured using Transwell chambers (Corning Inc., Corning, New York, NY, USA) with 8 μm-pore polycarbonate filters coated with Matrigel matrix (BD Biosciences, Bedford, MA, USA) as previously described (20). HT-29 cells were seeded at a density of 0.5×10^4^ cells/well in the upper compartment of each invasion chamber and incubated for 24 h in the absence or presence of 100 U/ml interferon (IFN)-γ (Sigma-Aldrich), 10 ng/ml interleukin (IL)1-α (PeproTech, Inc.) or 25 ng/ml tumor necrosis factor (TNF)-α (R&D Systems), plus extract of Cnidii Rhizoma (0.1–5 mg/ml) or 1400W (0.5 mM). Non-migrating cells on the upper surface of the membrane were gently scrubbed with a cotton swab, and the invading cells on the lower surface were fixed with 100% methanol and stained with hematoxylin and eosin Y solution (RICCA Chemical Company, Charlotte, NC, USA). The number of cells was counted under a microscope at a magnification of ×100.

### Gelatin zymography

A gelatin zymography assay was performed as previously described (20). The HT-29 cells were plated at a density of 5×10^6^ cells/well in 6-well plates. After 18 h, the monolayers were rinsed three times with phosphate-buffered saline followed by exposure to 100 U/ml IFN-γ, 10 ng/ml IL-1α and 25 ng/ml TNF-α, and extract of Cnidii Rhizoma (0.1–5 mg/ml) or 1400W (0.5 mM) under serum-free conditions for 24 h. The conditioned media was collected, normalized to the cell number, mixed with 10× non-reducing sample buffer (EZ BioResearch LLC, St Louis, MO, USA), consisting of 120 mM Tris-HCl (pH 6.8), 50% (v/v) glycerol, 4% (w/v) SDS, 28.8 mM 2-mercaptoethanol and 0.2% (w/v) bromophenol blue, and SDS-PAGE was subsequently performed using a gel containing 10% SDS and 0.1% (w/v) gelatin. The resulting gel was rinsed in 2.5% (v/v) Triton X-100 for 1 h and enzyme degradation was performed at 37°C for 18 h in 50 mM Tris-HCl (pH 7.5), 5 mm CaCl_2_ and 0.04% NaN_3_. The gel was subsequently stained for 30 min using 0.05% Coomassie Blue in 45% (v/v) methanol combined with 1% (v/v) acetic acid, and destained in a solution containing 10% acetic acid (v/v) and 25% methanol (v/v).

### Statistical analysis

The data were analyzed for statistical significance using Student’s *t*-test. P<0.05 was considered to indicate a statistically significant difference.

## Results and Discussion

### Induction of NO production in HT-29 cells

**Figure 1:**
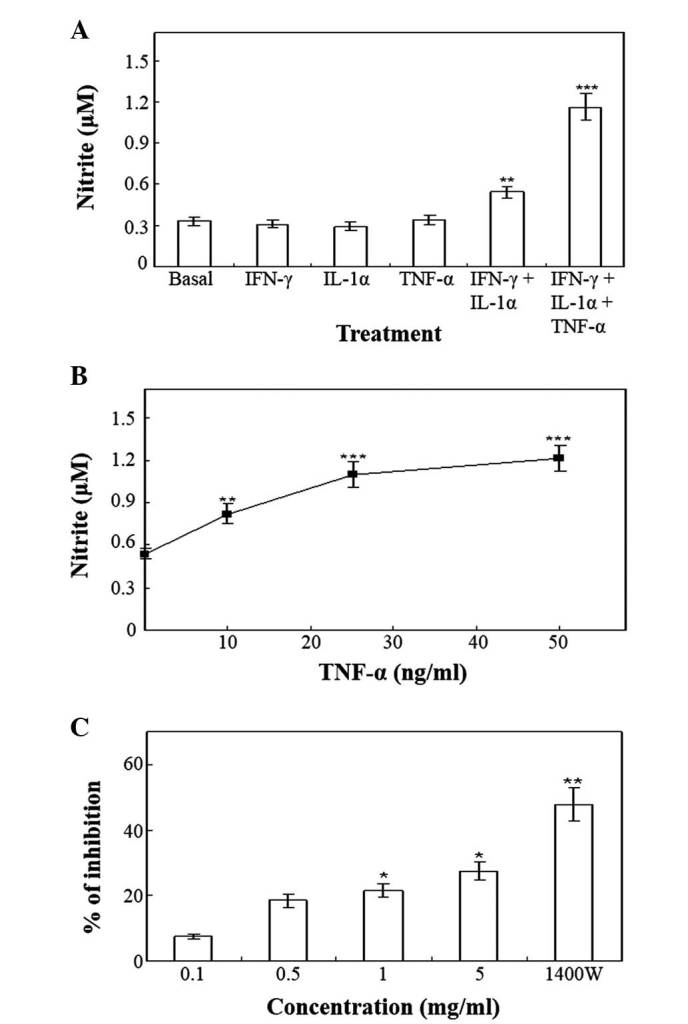
(A) Nitrite production by HT-29 cells following 48 h of treatment with cytokines. (B) Effect of 0–50 ng/ml TNF-α on nitrite production induced by 100 U/ml IFN-γ and 10 ng/ml IL-1α in HT-29 cells following 48 h of treatment. (C) Effect of water extract from Cnidii Rhizoma on cytokine-induced nitrite production in HT-29 cells following 48 h treatment with 100 U/ml IFN-γ, 10 ng/ml IL-1α and 25 ng/ml TNF-α. The data are presented as the mean ± standard deviation (n=3). ^*^P<.05, ^**^P<.01 and ^***^P<.005 vs. control. 1400W, 0.5 mM 1400W. IFN-γ, interferon-γ; IL1-α, interleukin-1α; TNF-α, tumor necrosis factor-α.

Upon stimulation with the vehicle for 48 h, the resting HT-29 cells produced basal levels of nitrite (Fig. 1A). The pro-inflammatory cytokines, IFN-γ (100 U/ml), IL-1α (10 ng/ml) and TNF-α (50 ng/ml), did not affect the production of nitrite when added alone to HT-29 cells (Fig. 1A). The minimum requirement for enhanced nitrite production was the combination of IFN-γ and IL-1α (Fig. 1A), while other combinations of cytokines were ineffective. The addition of TNF-α to the combination of IFN-γ and IL-1α produced an approximately two-fold enhancement in IFN-γ and IL-1α-induced nitrite production at 48 h (Fig. 1A). Various concentrations of TNF-α (0–50 ng/ml) in the presence of the combination of 100 U/ml IFN-γ and 10 ng/ml IL-1α, induced a concentration-dependent enhancement of nitrite production (Fig. 1B). Pretreatment of the cells with ≥5 mg/ml Cnidii Rhizoma or 0.5 mM 1400W, exerted an inhibitory effect on the production of nitrite induced by treatment with 100 U/ml IFN-γ, 10 ng/ml IL-1α and 25 ng/ml TNF-α (Fig. 1C). NO has been previously implicated in tumor biology. Previous studies have demonstrated that the expression level and activity of iNOS correlates with the histological grade of malignancy in human gynecological (21), breast (22), central nervous system (23) and lung cancers (24). iNOS activity and the resulting NO concentrations have been demonstrated to contribute to tumor progression by mediating tumor vascularization and tumor blood flow (21). iNOS was also induced in human colon adenocarcinoma, ovarian and glioblastoma cell lines in response to cytokine stimulation (21). The enhanced expression of iNOS in human colon carcinoma correlates with tumor growth and vascular invasion and may be indicative of the survival potential of cells (8).

### Induction of iNOS expression in HT-29 cells by a mixture of IFN-γ, IL-1α, and TNF-α

**Figure 2:**
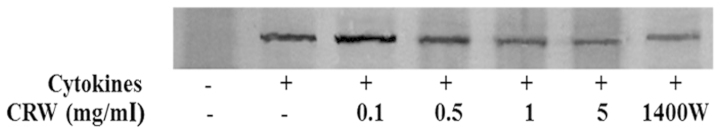
Inhibitory effect of water extract from Cnidii Rhizoma on the inducible nitric oxide synthase protein expression induced by 100 U/ml interferon-γ, 10 ng/ml interleukin-1α and 25 ng/ml tumor necrosis factor-α. CRW, water extract from Cnidii Rhizoma; 1400W, 0.5 mM 1400W.

Fig. 2 shows that the induction of iNOS expression in HT-29 cells by 100 U/ml IFN-γ, 10 ng/ml IL-1α and 25 ng/ml TNF-α. iNOS was not detected in the absence of the cytokines. Treatment of the cells with Cnidii Rhizoma reduced the expression of iNOS in a dose-dependent manner (Fig. 2). The present study also demonstrated that 1400W inhibited cytokine-induced iNOS expression.

### Invasiveness of HT-29 cells

**Figure 3:**
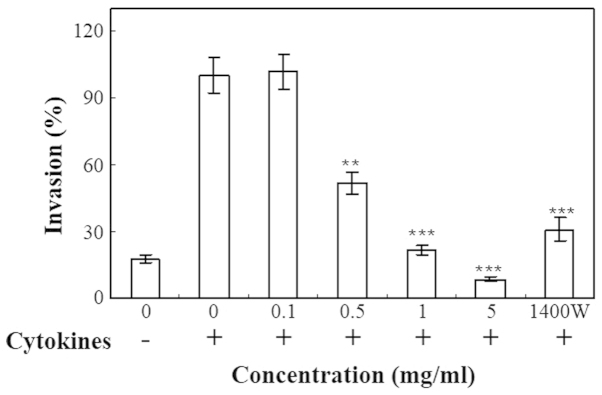
Effect of water extract from Cnidii Rhizoma on the invasiveness induced in HT-29 cells by 100 U/ml interferon-γ, 10 ng/ml interleukin-1α and 25 ng/ml tumor necrosis factor-α. The data are presented as the mean ± standard deviation (n=3). ^**^P<.01; ^***^P<.005 vs. control. 1400W, 0.5 mM 1400W.

Transwell plates were used to measure the invasive properties of cells following stimulation with 100 U/ml IFN-γ, 10 ng/ml IL-1α and 25 ng/ml TNF-α. The invasion of HT-29 cells through Matrigel was significantly increased by treatment with cytokines (Fig. 3). Cnidii Rhizoma inhibited the invasiveness of cytokine-treated HT-29 cells through the Matrigel-coated membrane in a concentration-dependent manner (Fig. 3). The invasiveness of cells was inhibited by treatment with 1400W, an iNOS inhibitor, which confirms that iNOS contributes to the process of tumor cell invasion. 1400W is an irreversible inhibitor of human iNOS, as well as a weaker and reversible inhibitor of human nNOS and eNOS. The potency and selectivity of 1400W to iNOS *in vitro* and *in vivo* is increased compared with any other described iNOS inhibitor (25). The present study demonstrated that 1400W inhibited NO production (Fig. 1C) and iNOS expression (Fig. 2) in HT-29 cells. Inhibition of NO production by 1400W was accompanied by a reduction of HT-29 cell invasion through the Matrigel (Fig. 3). The present study revealed that cytokine treatment increased NO production in HT-29 cells. Cytokines further enhanced the invasiveness of the HT-29 cells. The present results suggest that endogenous NO production induced by cytokines increases the invasion of the human colorectal adenocarcinoma HT-29 cells. In the HT-29 cell Matrigel assay, the inhibition of invasion caused by treatment with 1400W, the most selective iNOS inhibitor, demonstrated the involvement of iNOS. The cytokine-stimulated invasion of HT-29 cells was not completely abolished by 1400W, which indicates that other mechanisms may also induce invasion, in addition to the iNOS/NO pathway.

### MMP-2 activity in HT-29 cells

**Figure 4:**
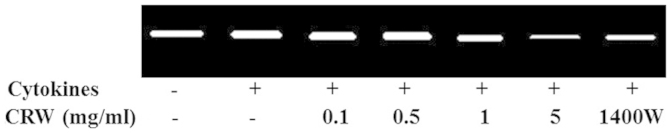
Effect of water extract from Cnidii Rhizoma on cytokine-induced MMP-2 activity in HT-29 cells. Gelatin zymography was used to detect MMP-2 activity in conditioned media obtained from HT-29 cells grown with cytokines (100 U/ml interferon-γ, 10 ng/ml interleukin-1α and 25 ng/ml tumor necrosis factor-α), cytokines plus water extract from Cnidii Rhizoma (CRW; 0.1–5 mg/ml) or 0.5 mM 1400W.

MMP-2 is one of the key enzymes involved in the degradation of the backbone of the cellular basement membrane, type IV collagen (26). To determine the activity of MMP-2, gelatin zymography was conducted using the conditioned medium, which was collected and measured following the treatment of cells for 24 h with the cytokines, and pretreatment with Cnidii Rhizoma or 1400W. MMP-2 activity in HT-29 cells was increased by the treatment of cytokines (Fig. 4). At a concentration of 5 mg/ml, Cnidii Rhizoma pretreatment inhibited cytokine-induced MMP-2 activity (Fig. 4). An association was observed between a decrease in MMP-2 levels in HT-29 cells and a reduction in invasiveness. MMP-2 activity was also inhibited by 1400W (0.5 mm), which indicates that iNOS contributes to the induction of MMP-2 activity (Fig. 4). MMPs are significant in tumor invasion and metastasis through the proteolysis of several extracellular matrix proteins and are overexpressed during tumor progression. The present study reveals that pro-inflammatory cytokines induce NO production, iNOS expression and the invasiveness of human colorectal adenocarcinoma HT-29 cells, whilst pretreatment with Cnidii Rhizoma inhibited these processes. The present results may provide sufficient information for the further development of Cnidii Rhizoma as an antitumor metastatic agent against colon cancer in animal studies and later in human clinical trials.

In conclusion, this study revealed that pro-inflammatory cytokines induce NO production, iNOS expression and invasiveness of human colorectal adenocarcinoma HT-29 cells. In addition, pretreatment with Cnidii Rhizoma inhibited cytokine-mediated NO production, iNOS expression and invasiveness of HT-29 cells. Therefore, future animal studies and subsequent human clinical trails are required to investigate the potential antitumor and antimetastatic effects of Cnidii Rhizoma against colon cancer. In addition, considering the designing of appropriate strategies for an intervention, further studies regarding the active components of Cnidii Rhizoma are also required to investigate the mechanisms regulated by Cnidii Rhizoma.
